# The Risk of Emerging of Dengue Fever in Romania, in the Context of Global Warming

**DOI:** 10.3390/tropicalmed8010065

**Published:** 2023-01-15

**Authors:** Larisa Maria Ivanescu, Ilie Bodale, Smaranda Grigore-Hristodorescu, Gabriela Martinescu, Bianca Andronic, Simona Matiut, Doina Azoicai, Liviu Miron

**Affiliations:** 1 Faculty of Veterinary Medicine, “Ion Ionescu de la Brad” University of Life Science, 8 Mihail Sadoveanu Alley, 700490 Iași, Romania; 2 Faculty of Horticulture, “Ion Ionescu de la Brad” University of Life Science, 8 Mihail Sadoveanu Alley, 700490 Iași, Romania; 3 Faculty of Medicine, “Grigore T. Popa” University of Medicine and Pharmacy, 16 Universității Street, 700115 Iași, Romania; 4 Praxis Medical Laboratory, 33 Independentei Boulevard, 700102 Iași, Romania

**Keywords:** *Aedes albopictus*, spatial distribution, climate conditions, dengue risk in Europe, bioclimatic indices

## Abstract

(1) Background: Few studies to date have assessed the influences induced by climate change on the spatial distribution and population abundance of *Aedes albopictus* using the latest climate scenarios. In this study, we updated the current distribution of *Ae. albopictus* mosquitoes and evaluated the changes in their distribution under future climate conditions, as well as the risk of dengue virus emergence in Romania. (2) Methods: Under the two scenarios: High scenario (HS) when no drastic measures to reduce the effects of global warming will be taken, or they are not effective and low scenario (LS) when very stringent greenhouse control measures will be implemented. (3) Results: The results estimate an increase in temperatures in Romania of up to 2.6 °C in HS and up to 0.4 °C in LS, with an increase in the period of virus replication within the vector from June to October in HS and from May to September in LS. Moreover, in 2022, *Ae. albopictus* was reported in a new county, where it was not identified at the last monitoring in 2020. (4) Conclusions: The rapid spread of this invasive species and the need to implement monitoring and control programs for the *Aedes* population in Romania are emphasized.

## 1. Introduction

Dengue fever is a neglected tropical disease representing a major public health concern [1], along with other diseases such as *Chagas*, leishmaniasis, *Chikungunya*, schistosomiasis, and others. The US Centers for Disease Control and Prevention estimate about 400 million diagnosed cases and up to 20,000 deaths from this disease [2,3]. Dengue fever is transmitted by the *Ae. Aegypti* and *Ae. Albopictus* mosquitoes, showing high fever with acute onset, joint and muscle pain, skin rashes, myalgias, hemorrhagic episodes, and circulatory shock [4,5]. Studies show that *Ae. albopictus* continues to spread, replacing *Ae. aegypti* in some areas, being an anthropophilic species. Consequently, it is important to review the literature and try to predict medical risks also in the context of global warming. Surveillance must be maintained on the vectorial role of *Ae. albopictus* in countries endemic for *dengue* and other arboviruses, for which it would be competent and ecologically appropriate to serve as a transmission vector.

*Ae. albopictus* eggs can remain viable in the environment for long periods of time due to their ability to become dormant through two processes of quiescence and diapause, a characteristic which contributes to their dispersal and hinders control actions [6], making it the most important species in the transmission and maintenance of dengue fever outbreaks, along with *Ae. aegypti*.

The transmission of dengue fever is complex, being influenced by temperature, precipitation, and the degree of urbanization. The reports presented by the WHO and other international health authorities emphasize that climate change is one of the main factors contributing to the rapid spread of dengue fever [7].

The Partnership for Dengue Control (PDC) proposed a three-step process: (1) A critical evaluation of vector control actions currently in use and those under development [8,9,10], (2) establishing a consensus of existing actions which work best, and (3) establishing ways to combine the best vector control actions, which were systematically defined in this process, with the use of dengue vaccines [11,12]. Thus, an effective measure to keep dengue fever under control is permanent surveillance of the vector population by using sampling methods for adults, such as vacuum cleaners [13,14], Biogents Sentinel traps, [7] methods which allow true measuring of adult *Ae.* populations. It is considered that the surveillance of adult female populations provides a much more complex picture of the impact of interventions on the risk of dengue virus contamination than the surveillance of immature mosquitoes [7,15]. Climatic factors are crucial determinants in the transmission of dengue fever by affecting the dynamics of its vectors [16,17]. Global and local climate not only influences the spatial distribution of infections [18] but also interannual variability [19]. The rapid change in climate factors in the last decade leads to an increase in the risk of dengue outbreaks [20]. An early warning system based on the calculation of climate factors would have the potential to improve disease surveillance and control. Studies show that the dengue fever virus is very sensitive to variations in climatic factors, temperature, and precipitation, which makes its transmission manifest on a local scale different from global expectations [21,22,23]. Thus, it is necessary to implement supported monitoring and control programs in limited areas, with the possibility to successfully predict the incidence of dengue through models determined by the climate [24,25].

## 2. Materials and Methods

### 2.1. Working Protocol

#### 2.1.1. Vectors

*Ae. albopictus* is a species that is very sensitive to temperature variations, with single cold extremes expected to persist in warming scenarios, which can have a very strong impact on reproductive success.

Laboratory studies have shown that the lowest temperature threshold tolerated by *Ae. albopictus* eggs was −10 °C (12 and 24 h), *Ae. albopictus* eggs without diapause in Europe hatch after treatment at −7 °C cold (8, 12 and 24 h exposure).

Lower temperature threshold tolerated by European *Ae. albopictus* eggs that have undergone a diapause was −10 °C for long-term exposure (12 and 24 h) and −12 °C for 1-h exposure. Eggs without diapause of *Ae. albopictus* in Europe hatch after treatment at −7 °C cold (8, 12, and 24-h exposure).

Thus, hatching success after cold treatment was significantly increased in European eggs that underwent diapause as compared to eggs without diapause. In nature, hatching of mosquito eggs to adult stage was calculated in the range of 10.5–36.8 °C, being a hardy species with incredible adaptability to new climatic conditions.

Studies show that at temperatures of 25 °C to 30 °C, a female of *Ae. albopictus* can lay about 70 eggs every 3 days in areas that can serve as shelter and contain water, such as old tires, holes in stones, various containers, gutters. The eggs, laid in isolation, can withstand periods of drought for several months, resuming their development at the first rain. The larval cycle lasts an average of eight days before reaching the adult stage, which lasts 4–6 weeks [26].

**Figure 1:**
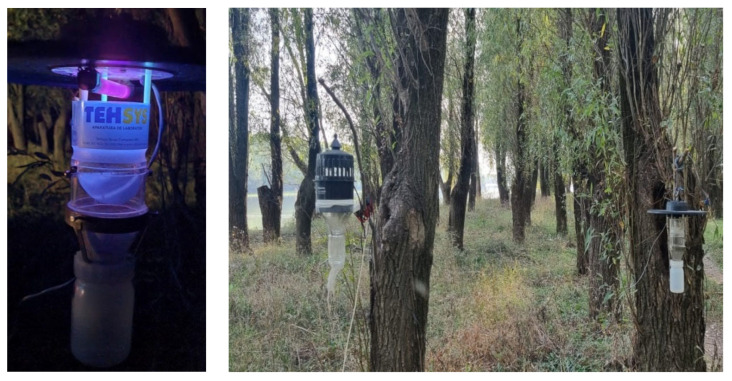
CDC Light Traps and the areas considered favorable for the development of the vector.

In the period April–October 2022, in the area of the Insula Mare a Brailei, on the Macini-Dunarea Veche branch, and in the Northeastern part of Romania in the Iasi City, in order to see if *Ae. albopictus* has a tendency to spread towards the north of the country, traps were placed in areas considered favorable for the development of the vector (trees with bark, water accumulations, abundant vegetation). CDC Light Traps (Figure 1) with dry ice as an attractant and hand aspirator were used to collect females directly from the human host. Traps were set in the morning and left until dusk. Females exhibit anthropophilic behavior and wean at dawn and dusk between 15:00 and 19:00. Mosquitoes were identified based on morphological characters using identification keys presented by Becker and interactive keys from MosKeyTool Version 2.2 (2020) software (Institut Pasteur, Paris, France).

In the Insula Mare a Brailei, the traps were placed in areas favorable for the hatching of eggs and the development of the larval stage, with many trees with hollows, but it is also an area populated by many fishermen, representing a host for the adult female *Ae. albopictus*. The Insula Mare a Brailei is an island on the Danube river in the Braila County, Romania, which has about 5000 inhabitants, but with an ideal habitat for the populations of *Ae. albopictus.* Being a tourist area offering fishing spots, people from the whole country come here, representing a source of dissemination in the territory. I chose this region because it is adjacent to the capital Bucharest, where the species *Ae. albopictus* was reported for the first time, and most cases of imported dengue fever were diagnosed here. Many people from Bucharest go to the Big Island of Braila for recreation and, especially, to practice fishing.

The Iasi County is located in the Central-Eastern of Moldavia and Northeastern area of Romania. The traps were placed in three areas of the city of Iasi: the Cotu Morii pond (a pond situated 20 km from Iasi, on the Iasi-Sculeni road, near human dwellings. The water is relatively clean, with abundant vegetation), the Ciurbesti natural lake (situated at 192 m altitude, with relatively clean and abundant vegetation) and the Nicolina River (Galata district) the riverbed is dirty and has abundant vegetation, the water being polluted by the industrial units which are on the route, as well as by the household waste. The river is near the Rotunda hill at an altitude of over 350 m, and it flows into the Bahlui River on the territory of the municipality of Iasi). The Municipality of Iasi is the most populous metropolitan area in Romania, and it is one of the most important education and research centers in the country, accommodating over 60,000 students in five public universities, with many foreign students who come from regions endemic for dengue fever.

#### 2.1.2. Virus

It should be noted that the dengue virus (DENV) can cause a number of manifestations, such as dengue hemorrhagic fever (DHF), dengue fever (DF), and dengue shock syndrome (DSS), with approximately 96 million cases and more than 390 thousand people diagnosed showing clinical symptoms, which are all the more serious in a population with a total lack of immunity. The severity of cases is even greater in the population of non-endemic countries.

**Figure 2:**
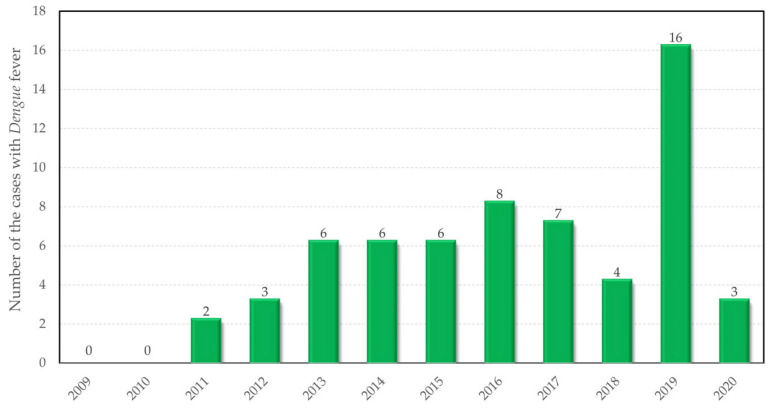
The number of imported cases of dengue fever registered in Romania between 2009 and 2020.

In Romania, so far, all diagnosed cases of dengue fever have been imported, in the 12 years considered, 61 cases have been registered, with a large increase in 2019 (Figure 2). The fact that every year there have been diagnosed cases of dengue fever underlines the primary need for monitoring the vector and climatic conditions in order to prevent the emergence of indigenous cases. Thus, the study aimed to determine the risk of dengue fever transmission in Romania, given the existence of the vector in the wild and the existence of favorable climatic factors [27].

#### 2.1.3. Measurement of Climatological Parameters

The air temperature was measured in the Weather Shelter, at the height of 2 m above the ground, four times a day during the observation hours, and precipitation was recorded with a rain gauge at the height of 1.5 m, according to World Meteorological Organization (WMO) standards. The measurements were made by the National Meteorological Administration (ANM), and they were reported in the European Climate Assessment and Dataset [28]. The results were recorded by several meteorological stations in regions with low relief forms: Muntenia (South of Romania), Moldova (East of Romania), Transylvania (Central and West of Romania), Oltenia (South-West of Romania), and Danube Meadow (South of Romania).

In order to obtain the most accurate data possible on the phenomena monitored, the results recorded by several meteorological stations in the same region were used. The development of *Ae. albopictus* populations and the capacity of the dengue virus to replicate inside the mosquito were monitored. For this purpose, we divided Romania into five regions: Muntenia (with stations in Bucharest-Baneasa, Rosiorii de Vede, Calarasi, Buzau), Moldova (with meteorological stations in Botosani, Iasi, Bacau), Transylvania (with stations in Cluj-Napoca, Sibiu, Arad), Oltenia (Ramnicu Valcea, Drobeta Turnu Severin, Craiova), and the Danube Meadow (with stations in Calarasi, Galati, Tulcea).

#### 2.1.4. Bioclimatic Indices

To be able to prevent future dengue epidemics in Romania, it is necessary to establish the bioclimatic conditions which can cause the occurrence of dengue outbreaks. In this sense, we studied the climate changes in several regions in Romania to establish the areas with potential risks. In this study, we proposed the use of bioclimatic indices to estimate the favorable periods for the development of *Ae. albopictus* mosquito populations and the incubation periods of the virus inside the vector.

- Potential development period index of *Ae. albopictus* (MPI)

We introduced into the study the potential development period index of *Ae. albopictus* (MPI) as an indicator of potential mosquito development periods according to temperature conditions. This index takes into consideration all monthly possible periods (MPIm) computed as the sum of optimal temperature required by mosquito larvae to reach maturity (Equation (1)):

(1) $$\;\mathrm{MPI}=\overset{12}{\underset{m=1}{\sum }}{\mathrm{MPI}}_{m}$$

In the present study, we aimed to determine the population of *Ae. albopictus*, in terms of the risk of transmission of the dengue virus, by calculating the number of days favorable for the formation of the mosquito population, in ideal conditions with temperatures of at least 27 °C (a complete cycle from egg to adult is 7 days), which also coincides with a favorable period for the replication of the virus inside the mosquito, which can contaminate the host on which it feeds, causing the disease. The index is important to calculate the maximum possible number of eggs per year under ideal local temperature conditions.

- Potential Infestation Index of dengue virus (PII)

The second index defined by us is the potential index of infestation with the dengue virus (PII), which calculates the maximum possible periods from the infection of female mosquitoes to the first day when they can transmit the virus. The maximum possible annual periods are 24 h at a temperature above 27 °C. The Potential dengue Infestation Index (PII) is calculated as the maximum possible thermal ranges from all months, favorable for infection with the dengue virus (Equation (2)):

(2) $$PII=\overset{12}{\underset{m=1}{\sum }}PII_{m}$$

The bioclimatic indices proposed are a useful method for estimate the evolution of the thermal conditions favorable for developing the mosquito populations and periods of infection with the dengue virus in different regions from Romania. Estimation of temperatures in 2100.

In recent years, several models have been proposed to estimate temperatures for the next decades. For the year 2100, we simulated average daily temperatures to make a forecast of temperature changes relative to the climatological reference period 1981–2000. These simulations were carried out using the long-term scenarios of the Fifth Assessment Report (AR5) of Working Group III of the Intergovernmental Panel on Climate Change (IPCC). The series of “representative concentration pathways” (RCP) models estimate changes in greenhouse gas concentrations based on policies implemented by states in the coming years. Predictions are based, according to this approach, on two estimate scenarios regarding the temperature’s projection in 2100. We considered the optimist case, called low scenario (LS), which corresponds to the RCP 1.9 scenario of Coupled Model Intercomparison Project Phase 5 (CMIP5) and indicates an increase in temperatures, in the Northern Hemisphere, on average of by 1.5 °C, if tough measures are taken to limit greenhouse gas (GHG) emissions, and undesirable scenario, in which it will be the higher temperature on the ground, defined as the high scenario (HS). In this case (RCP 8.5), the temperatures will increase in 2100 by 4.5 °C compared to the pre-industrial era. HS corresponds to a scenario in the absence of specific actions to control greenhouse gas emissions or if they are not effectively implemented [28,29]. The simulations show that global temperature would have a linear trend at least till 2100 in all scenarios of CMIP5.

## 3. Results

The last study conducted in Romania on the distribution of *Ae. albopictus* mosquitoes was carried out in 2020 by Fălcuta et al. [30] when the invasive species was reported in Covasna, Sibiu, Bihor, Prahova, Ilfov, Giurgiu, and Constanta counties. In this study, we considered a neighboring county to Ilfov County (where this species was reported for the first time and where most cases of imported dengue fever were diagnosed), Braila County, in the south-eastern part, where this invasive species has not been reported in the past, and we also considered the Iasi City from North-East of Romania.

**Figure 3:**
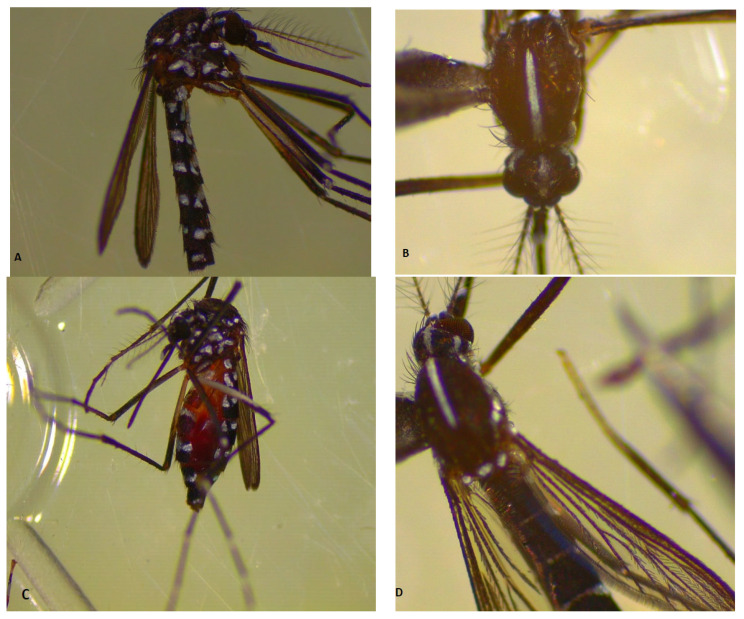
*Ae. albopictus*: (**A**) Unfed female, (**B**) specific morphological feature of the thorax, (**C**) fed female, (**D**) specific morphological characters: Head, thorax, and abdomen.

The species *Ae. albopictus* has been identified in the Big Island of Braila, on the Macini-Dunarea Veche branch, most of the females being caught with a hand aspirator, directly from the human host, in the 14–16°° range, showing an increased aggressiveness of the females (Figure 3).

The mathematical calculations used by us show that if drastic measures are taken to limit greenhouse gas emissions, temperatures will increase by 0.4 °C in the region of Moldova by 2100 (Low Scenario—LS) and by 2.6 °C (High Scenario—HS) if these specific actions are not taken, or not taken efficiently. Dengue virus will replicate within the vector, establishing a transmission season from July to September under LS conditions, and from June to September with an increase in the number of days under HS conditions, compared to the current period, when until 2020, the longest interval favorable for virus replication was recorded five times.

For the Romanian Plain, temperatures are expected to increase by 0.4 °C in LS and by 2.6 °C in HS until 2100, but with an increase in the period of virus replication from June to October in HS, and from May to September in LS.

The same temperature change can also be observed in the Oltenia region, with a prolongation of the risk of virus transmission in October in HS. In the Danube Delta, an increase in temperatures by 2100 in LS by 0.3 °C and in HS by 2.5 °C is observed, and the risk of Dengue virus transmission increases to 38 favorable periods from June to October in HS. In Transylvania, the temperature will increase by 0.2 °C in LS and by 2.4 °C in HS by 2100, with a 6-fold risk of virus transmission from July to September (Table 1).

Regarding the favorable periods for the development of *Ae. albopictus* populations, it can be observed that in the Danube Valley and in Oltenia Plain, from March to November, there was at least one favorable period for the development of a complete cycle from egg to adult.

**Figure 4:**
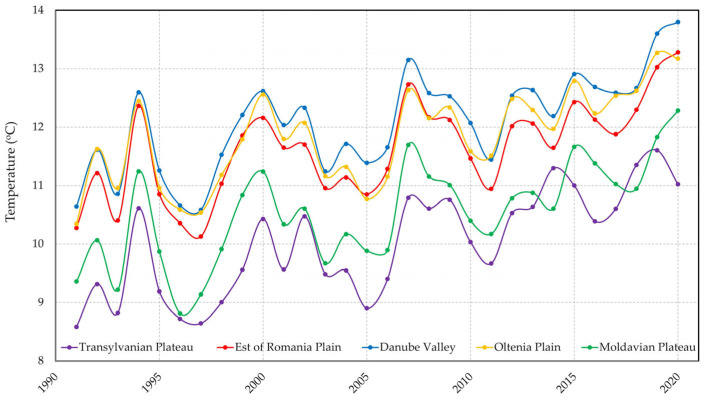
Temperature evolution in Romania during 1990–2020.

The average annual temperatures recorded at meteorological stations in Romania in the period 1990–2020 have shown a steady increase since 1991 (Figure 4). Temperature records were recorded in the years 2019–2021, years that proved to be equally important globally, with a significant impact on the oceans, atmosphere, cryosphere, and surface temperature of the planet. In 2021 globally, it was the hottest Northern Hemisphere summer (June, July, and August) recorded on Earth, along with extreme heat waves, wildfires, and precipitation.

**Figure 5:**
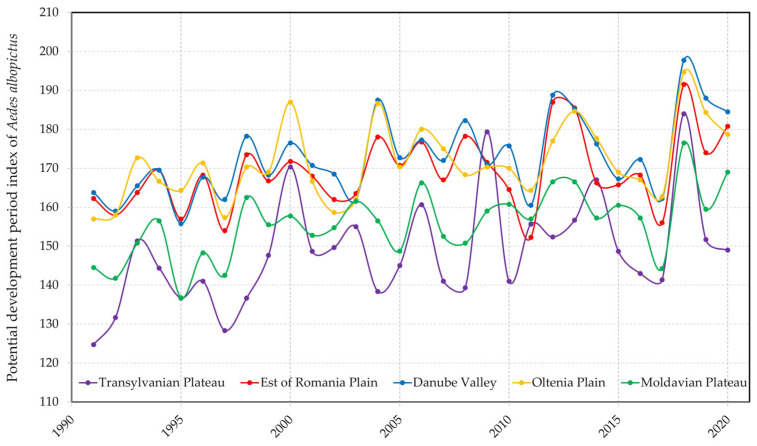
The development of the *Ae. albopictus* populations.

In the graph in Figure 5 it can be seen an increase in the periods favorable to the development of the *Ae. albopictus* population, as in 2019, there were almost 200 days favorable to the evolution of the biological cycle, and in the same year, there was a record number of imported cases of dengue fever. The need for permanent monitoring of the mosquito population, as well as the association of the evolution of climatic factors, in order to tighten the vector control actions and prepare the medical framework for the eventual occurrence of autochthonous cases is thus signaled. The results of the influence of climatic factors on the development of the vector population, as well as the risk of virus replication within the vector, show a favorable modification of both processes taken into account, requiring the implementation of rigorous monitoring and control programs.

**Figure 6:**
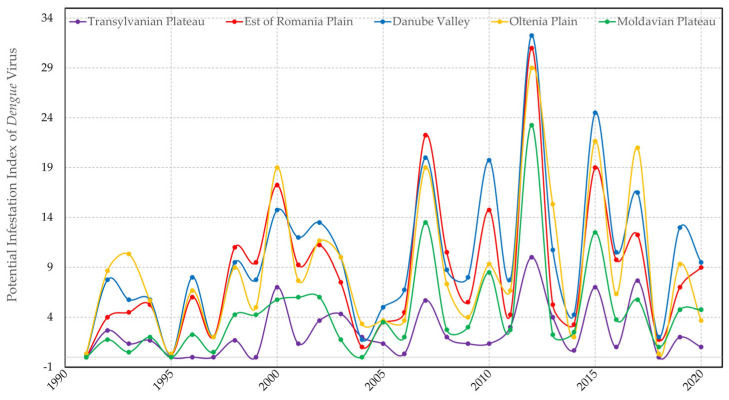
Periods of dengue virus replication inside the vector.

A study conducted by Liu et al. in 2017 by experimentally infecting *Ae. albopictus*, with dengue virus 2 (DENV-2) and then exposing it to constant temperatures (18, 23, 28, and 32 °C) and a fluctuating temperature (28-23-18 °C) demonstrated that at 18 °C, the virus developed slowly in the midgut of the vector, without spreading to the salivary glands. At 23 and 28 °C, the virus was detected in the salivary glands and ovaries 10 days post-infection. Rates of dissemination, infection, and transmission in humans were higher at 27 °C, (7–8 days) compared to 23 °C and at this temperature the amount of virus in salivary glands can cause the disease in humans. At 32 °C, the incubation period of the dengue virus inside the mosquito was of only five days [31]. We calculated the evolution of the risk of dengue virus transmission from 1990 to 2020 (Figure 6), taking into account the periods of the year when seven consecutive days with temperatures above 27 °C were recorded. However, by the year 2100, under High Scenario conditions, the periods which ensure the transmission of the dengue virus through its replication and concentration at the level of the salivary glands will triple throughout the country.

**Figure 7:**
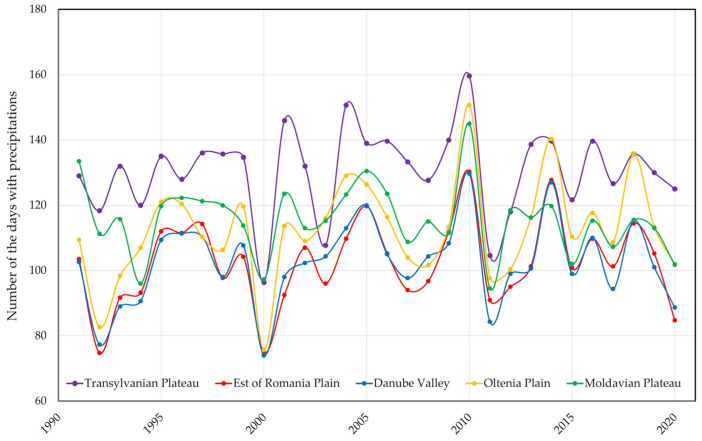
Days with precipitation in the period 1990–2020.

While temperature influences mosquito reproduction, maturation, and mortality rates, rainfall leads to the formation of breeding sites for larvae and pupae. Therefore, studies suggest that winter rains may cause mosquito eggs to hatch before spring, which could cause larvae to die as a result of low temperatures [32]. The graph from Figure 7 represents the number of days with precipitation in all areas of the country where the temperatures were calculated. *Ae. albopictus* is a species that needs water for the eggs to hatch, but if precipitation occurs in winter, the hatched larvae do not survive. In the graph represented, it can be seen that a higher number of days with precipitation was recorded in the Moldova area, the area located in the Northeastern part of Romania, the area where the present *Ae. albopictus* species was not reported. It seems that even if we have rainy seasons favorable for egg hatching, higher temperatures are more important, necessary for the development of the other stages of development. Eggs of *Ae. albopictus* are very resistant to drought, which allows survival in drought conditions and the development of large populations when the climatic factors are favorable.

## 4. Discussion

The detection of the species *Ae. albopictus* in a new county is an alarm signal on the need to introduce mandatory monitoring and control programs for the *Ae.* population in the context of global warming, as there is a risk of emergence of vector-borne diseases in Romania. Temperatures can be a strong ecological constraint. The climate change predicted for the winter season shows an increase in average minimum temperatures in the coldest winter quarter, with frosty days becoming less frequent, results that seem to be valid for the whole of Europe [26].

In this context, we followed the evolution of temperatures in Romania until 2100 to estimate the possible risk of dengue virus transmission in regions favorable to the development of mosquitoes.

In the Danube Meadow and East of the Romanian Plain, from the point of view of temperatures, the populations of *Ae. Albopictus* can develop from March to November, but the dengue virus can replicate in the salivary glands and can produce the disease during the feeding of the female mosquito, only in the months of July, August, and September, especially in August. However, until the year 2100 in HS, the risk period of virus transmission increases to five months a year, from June to October.

These results provide important details to better guide the surveillance and control programs of *Ae. albopictus* population, also having significant importance for public health as a reference point for predicting the occurrence of dengue fever in Romania.

To date, numerous studies have been conducted on the global distribution of human dengue cases, but distributions along with key vector species have not been fully incorporated into these mapping efforts. The need for such studies in the conditions in which climate changes could produce distributional changes that, although not considerable, will have significant implications for public health worldwide is paramount. The use of the ROC and RCP model is preferred internationally in establishing the influence of climate change on the distribution of vector-borne diseases, such as dengue [33,34].

Most of the existing data are contained in institutional, national, and even personal research databases and are not available to the wider community, so carrying out more studies using such models and correlating the results would allow obtaining a global picture of the distribution of fever dengue reported to the *Ae. albopictus* vector.

A study done in 2019, using the geospatial projections of a mechanistic temperature-dependent transmission model, investigated the seasonal, geographic, and population changes of *Ae. albopictus*, in the risk of global virus transmission with climate warming by 2080, showing that the largest population increases are constantly projected in Europe. The current response to curb carbon emissions and keep temperatures below the 2 °C warming target, being insufficient, models such as those used can be useful as a means of anticipating temperature-driven transmission risk and possible future climate change depending on the degree of mitigation achieved [34].

The period from ingestion of dengue virus-infected blood until the disease can be transmitted by an *Aedes* mosquito female (extrinsic incubation period, EIP) is a very important factor in determining the vector capacity and transmission rate of the virus; therefore, it is not sufficient for the virus to be present inside the mosquito, it must have a sufficient replication period to be able to produce the disease. We point out that the calculations were made under ideal conditions of recording temperatures of at least 27 °C for seven consecutive days, emphasizing that the information obtained represents safe periods of Dengue virus transmission, periods in which drastic measures should be taken to control the *Ae. albopictus* population. 

Similar studies have shown that *Ae. albopictus* can transmit dengue fever with similar viral loads in mosquito saliva at 28 °C, underlining the major importance of temperatures in the risk of dengue outbreaks and the need for permanent monitoring of dengue cases related to climatic factors [35]. In Romania, there is no monitoring and control program for vectors and vector-borne diseases, considering until now that climatic factors have not favored the transmission of viruses. However, the cases of neuroinfection caused by West Nile infection have increased greatly, with a record in 2018. *Ae. albopictus* is an anthropophilic species that feeds during the day, with a biological cycle very different from that of the genus *Culex*, which is involved in the transmission of the *West Nile* virus. The spread of this invasive species in new territories must be considered when we talk about *West Nile*. In addition, as a result of the intensification of intercontinental travel, the number of imported cases of dengue fever has increased in Romania in recent years, making it mandatory to introduce programs to monitor vectors and vector-borne diseases. The study comes with an alarm signal in this sense, demonstrating the change of climate also in Romania, with a very important impact on the transmission of tropical vector diseases. To prevent and control an epidemic of dengue fever, it is mandatory to permanently monitor the mosquito population, to quickly diagnose cases of the disease, and, very importantly, both related to the evolution of climatic factors.

## 5. Conclusions

Global warming and increased intercontinental travel pose a risk of disease emergence in Romania, dengue fever being one of them. Increasing temperatures thus widen the geographical range of the vector, increase the number of blood samples, increase reproduction rates and shorten the incubation period of the pathogen inside the vector.

For a disease to become emergent in a given territory, three factors must coexist: a. The existence of the pathogen in nature, b. the existence of the vector in nature, c. the existence of favorable climatic factors to the development of the vector and the pathogen within it.

We have carried out an extrapolation of the temperature evolution for the year 2100, using the mathematical model, suggesting the progression of some favorable conditions for the development of the *Aedes* vector and of the dengue virus inside of it. The extrapolation of temperatures for the year 2100 shows an increase of temperatures by 2.6 °C in HS and 0.4 °C in LS. Consequently, in Romania, we have the *Ae. albopictus* vector in a continuous spread, we have annual cases of dengue fever import, and we have favorable climatic conditions. The risk of dengue fever epidemics in Romania is continuously increasing.

According to a study carried out in 2021, which made a prediction for the next 30 years, it showed that 68% of the European continent will become favorable for the development of *Ae. albopictus* populations, including most of the British islands, Ireland, and the southern areas of the Scandinavian countries [36].

Combining larger spatial models with smaller scale models of attack rates or outbreak size is essential to establishing a consensus and revealing the differences and similarities between all available models through transparency, having major importance in obtaining a global reality.

Densely populated urban areas favor the emergence of mosquitoes through the greenhouse effect, amplifying the effects of climate change with increasing urban temperatures and by providing breeding sites for mosquitoes in containers, artificial water, and irrigation. Therefore, measures to combat mosquitoes should be intensified in urban areas and always related to climatic factors, especially in big cities where cases of imported dengue have been reported. The Insula Mare a Brailei is an island on the Danube river in the Braila County, Romania, which has about 5000 inhabitants, but with ideal habitat for the populations of *Ae. albopictus.* Being a tourist area, offering fishing spots, people from the whole country come here, representing a source of dissemination in the territory. The results of our study underline the necessity of mandatory introduction of *Ae. albopictus* population monitoring programs in each county. The area covered by us is much too small, and the reality can be much worse.

The identification of the species *Ae. albopictus* in a new area shows the ability of this invasive species to spread quickly and the need for continuous monitoring to prevent the occurrence of dengue outbreaks if the vector spreads in Iasi areas, which is one of the most important education and research centers of the country with many foreign students who come from countries endemic for dengue fever.

## Figures and Tables

**Table 1:** Bioclimatic and temperature indices for 1991–2020 and estimates for 2100.

Region	Index	January	February	March	April	May	June	July	August	September	October	November	December	Annual
Moldavian Plateau	*T*_month_ (average 1991–2020)	−1.9	0.2	5.1	11.4	16.8	20.8	22.6	22.0	16.7	10.6	5.3	0.0	10.8
	*T*_month_ (estimation 2100—LS)	−0.5	1.2	4.8	11.7	17.3	21.4	23.0	22.2	16.6	11.4	4.8	0.2	11.2
	*T*_month_ (estimation 2100—HS)	1.7	3.4	7.0	13.9	19.5	23.6	25.2	24.4	18.8	13.6	7.0	2.4	13.4
	MPI_m_ (1991–2020)	0	0	0	9	27	30	31	31	23	5	0	0	156
	PII_m_ (1991–2020)	0	0	0	0	0	0	1	2	2	0	0	0	4
	PII_m_ (estimation 2100—LS)	0	0	0	0	0	0	1	3	3	0	0	0	6
	PII_m_ (estimation 2100—HS)	0	0	0	0	0	1	5	9	7	0	0	0	23
East of Romanian Plain	*T*_month_ (average 1991–2020)	−1.2	1.4	6.4	12.1	17.5	21.7	23.9	23.6	18.1	11.8	6.3	0.7	11.9
	*T*_month_ (estimation 2100—LS)	0.1	1.9	6.2	12.7	18.2	22.7	24.4	23.7	18.1	12.5	5.9	0.8	12.3
	*T*_month_ (estimation 2100—HS)	2.3	4.1	8.4	14.9	20.4	24.9	26.6	25.9	20.3	14.7	8.1	3.0	14.5
	MPI_m_ (1991–2020)	0	0	0	12	29	30	31	31	27	9	1	0	169
	PII_m_ (1991–2020)	0	0	0	0	0	0	1	4	3	0	0	0	8
	PII_m_ (estimation 2100—LS)	0	0	0	0	0	0	3	7	5	0	0	0	15
	PII_m_ (estimation 2100—HS)	0	0	0	0	0	1	8	15	12	1	0	0	37
Oltenia Plain	*T*_month_ (average 1991–2020)	−0.2	2.1	6.9	12.6	17.4	21.4	23.5	23.3	18.0	12.0	6.5	1.2	12.1
	*T*_month_ (estimation 2100—LS)	0.8	2.8	6.8	12.8	18.0	22.3	24.0	23.7	18.1	12.8	6.3	1.5	12.5
	*T*_month_ (estimation 2100—HS)	3.0	5.0	9.0	15.0	20.2	24.5	26.2	25.9	20.3	15.0	8.5	3.7	14.7
	MPI_m_ (1991–2020)	0	0	1	13	28	30	31	31	27	10	1	0	171
	PII_m_ (1991–2020)	0	0	0	0	0	0	1	4	4	0	0	0	9
	PII_m_ (estimation 2100—LS)	0	0	0	0	0	0	2	5	6	0	0	0	13
	PII_m_ (estimation 2100—HS)	0	0	0	0	0	1	7	13	12	1	0	0	34
Danube Valley	*T*_month_ (average 1991-2020)	−0.2	2.0	6.7	12.4	17.8	22.1	24.3	23.8	18.4	12.3	7.0	1.6	12.4
	*T*_month_ (estimation 2100—LS)	0.9	2.6	6.3	12.8	18.4	22.7	24.6	23.9	18.4	13.0	6.6	1.7	12.7
	*T*_month_ (estimation 2100—HS)	3.1	4.8	8.5	15.0	20.6	24.9	26.8	26.1	20.6	15.2	8.8	3.9	14.9
	MPI_m_ (1991–2020)	0	0	1	12	29	30	31	31	28	10	1	0	173
	PII_m_ (1991–2020)	0	0	0	0	0	0	2	4	4	0	0	0	10
	PII_m_ (estimation 2100—LS)	0	0	0	0	0	0	2	6	6	0	0	0	14
	PII_m_ (estimation 2100—HS)	0	0	0	0	0	1	8	16	12	1	0	0	38
Transylvania Plateau	*T*_month_ (average 1991–2020)	−1.7	0.6	5.2	11.1	15.8	19.5	21.2	20.9	15.7	10.3	5.1	−0.1	10.3
	*T*_month_ (estimation 2100—LS)	−0.8	0.6	4.9	11.0	16.1	19.8	21.3	21.1	15.8	10.8	5.1	0.1	10.5
	*T*_month_ (estimation 2100—HS)	1.4	2.8	7.1	13.2	18.3	22.0	23.5	23.3	18.0	13.0	7.3	2.3	12.7
	MPI_m_ (1991–2020)	0	0	0	9	23	30	31	30	21	5	0	0	149
	PII_m_ (1991–2020)	0	0	0	0	0	0	0	1	1	0	0	0	2
	PII_m_ (estimation 2100—LS)	0	0	0	0	0	0	0	1	2	0	0	0	3
	PII_m_ (estimation 2100—HS)	0	0	0	0	0	0	2	4	4	0	0	0	11

## Data Availability

Not applicable here.
